# Wallenberg syndrome in a patient after pulmonary resection: a case report

**DOI:** 10.1186/s44215-023-00065-y

**Published:** 2023-08-16

**Authors:** Tsubasa Matsuo, Daisuke Kimura, Kengo Tani, Takahiro Sasaki, Masahito Minakawa

**Affiliations:** Department of Thoracic and Cardiovascular Surgery, Graduate School of Medicine, Hirosaki University, Hirosaki City, Aomori, Japan

**Keywords:** Lung cancer, Wallenberg syndrome, Left upper lobectomy, Cerebral infarction

## Abstract

**Background:**

Cerebral infarction after pulmonary resection is a minor but critical complication. We report a rare case of postoperative complication of Wallenberg syndrome caused by cerebral infarction in the posterior inferior cerebral artery after the left upper lobectomy.

**Case presentation:**

A 72-year-old man developed cerebral infarction 2 days after a left upper lobectomy for lung cancer. Magnetic resonance imaging indicated right vertebral artery occlusion following an early ischemic area on the right lateral side of the medulla oblongata and cerebellum. Contrast-enhanced computed tomography revealed no thrombus in the left superior pulmonary vein stump. The patient was diagnosed with Wallenberg syndrome, and prompt anticoagulation therapy was initiated. The patient was discharged and transferred to another hospital for rehabilitation on postoperative day 16.

**Conclusions:**

We present a rare case of Wallenberg syndrome occurring in the posterior inferior cerebral artery area due to vertebral artery occlusion after lobectomy. Because cerebral infarction of the posterior circulation has many similar symptoms due to the side effects of anesthetic drugs, careful physical examination is required to determine Wallenberg syndrome.

## Background

Cerebral infarction is a rare but critical complication of pulmonary resection. Thrombus at the pulmonary vein stump or the left atrial appendage following atrial fibrillation often leads to cerebral infarction in the area of the middle cerebral artery. The causes of cerebral infarction are divided into thrombosis and embolism; most cerebral infarctions caused by embolism occur in the anterior cerebral circulation from the middle cerebral artery. Herein, we report a rare case of postoperative cerebral infarction representing Wallenberg syndrome that occurred in the posterior inferior cerebral artery due to vertebral artery occlusion.

## Case presentation

**Fig. 1:**
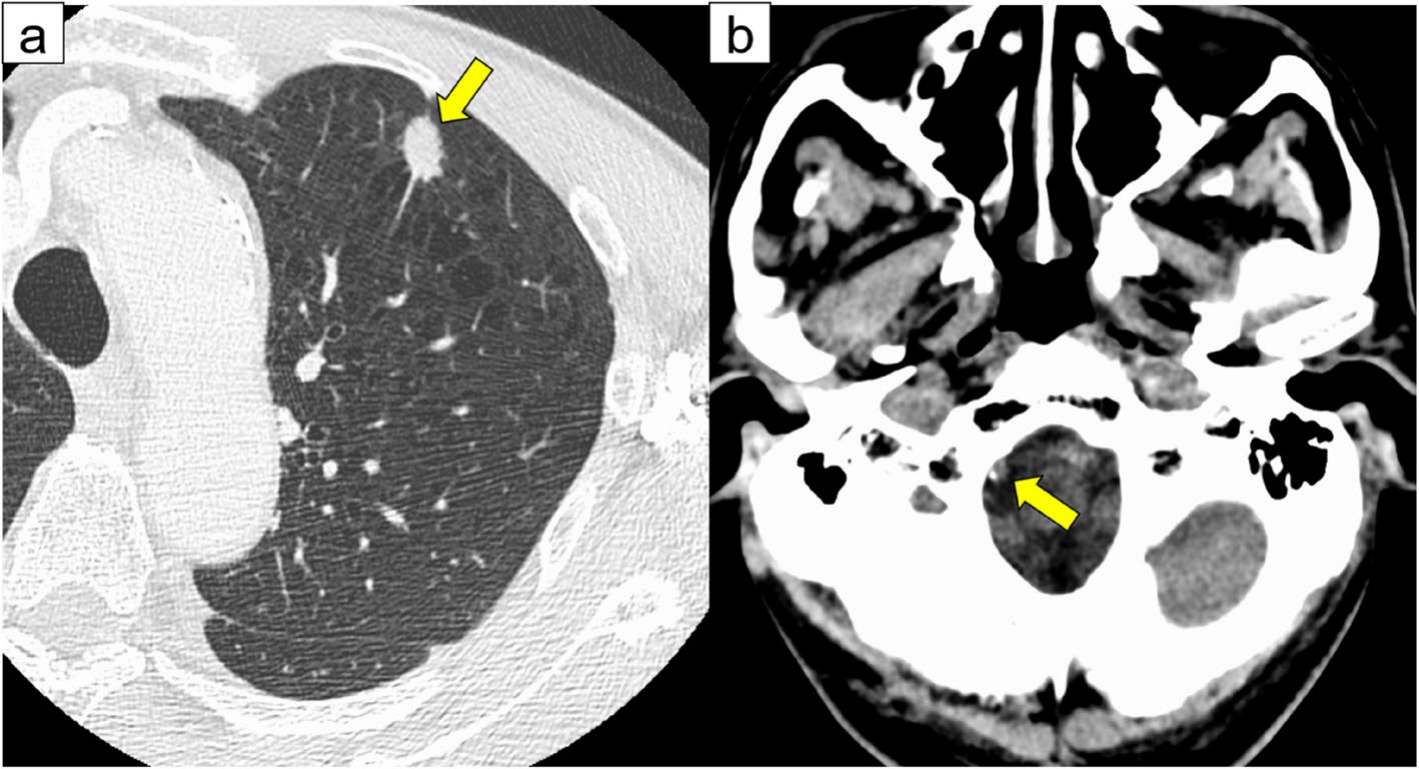
**a** Chest computed tomography scan showing a tumor in the left upper lobe of the lung (yellow arrow). **b** Head computed tomography scan showing slight calcification in the right vertebral artery (yellow arrow)

The patient was a 72-year-old male. He had a history of hypertension, chronic obstructive pulmonary disease, pneumothorax, and appendix cancer. He had smoked 30 cigarettes daily for 50 years but had no history of arrhythmia or cerebrovascular disease. His family doctor identified an abnormal shadow on the chest radiograph. Computed tomography (CT) revealed a tumor in the left upper lobe of the lung with no hilar or mediastinal lymph node swelling (Fig. 1a). Head CT showed slight calcification in the right vertebral artery (Fig. 1b). An 18-fluoro-2-deoxyglucose-positron emission tomography scan revealed that the maximum standardized uptake value at the center of the tumor was 10.4. CT-guided biopsy confirmed the diagnosis of lung adenocarcinoma (cT1bN0M0, cStageIA2, as defined by the 8th Edition of TNM classification of lung cancer).

**Fig. 2:**
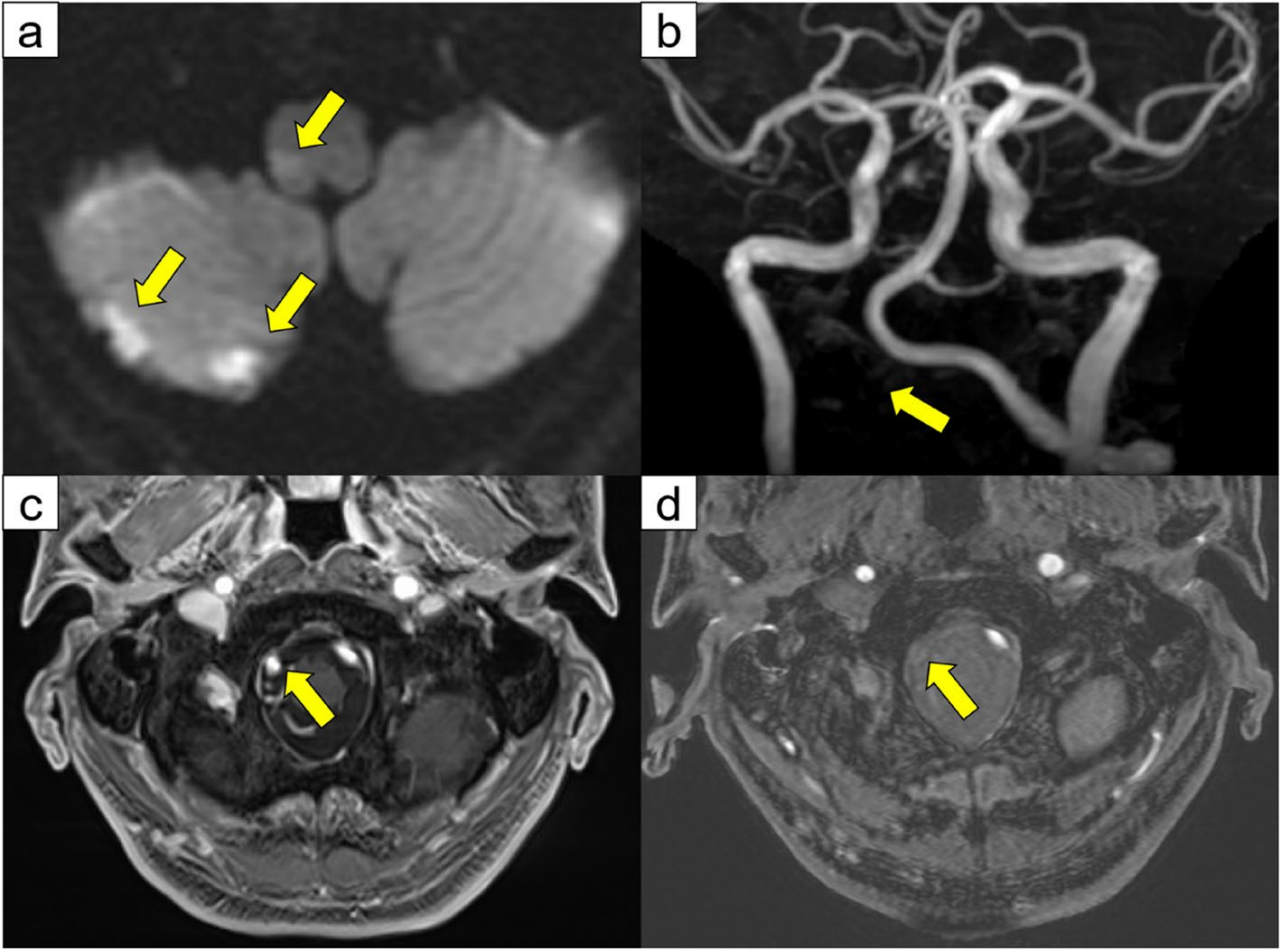
**a** Diffusion-weighted magnetic resonance image of the brain showing high-intensity signals in the medulla oblongata and the right cerebellum (yellow arrows). **b** Magnetic resonance angiography indicated occlusion of the right vertebral artery (yellow arrow). **c** The right vertebral artery can be seen on the preoperative head magnetic resonance image (yellow arrow). **d** Magnetic resonance imaging at the onset of cerebral infarction indicating a loss of signal in the right vertebral artery (yellow arrow)

**Fig. 3:**
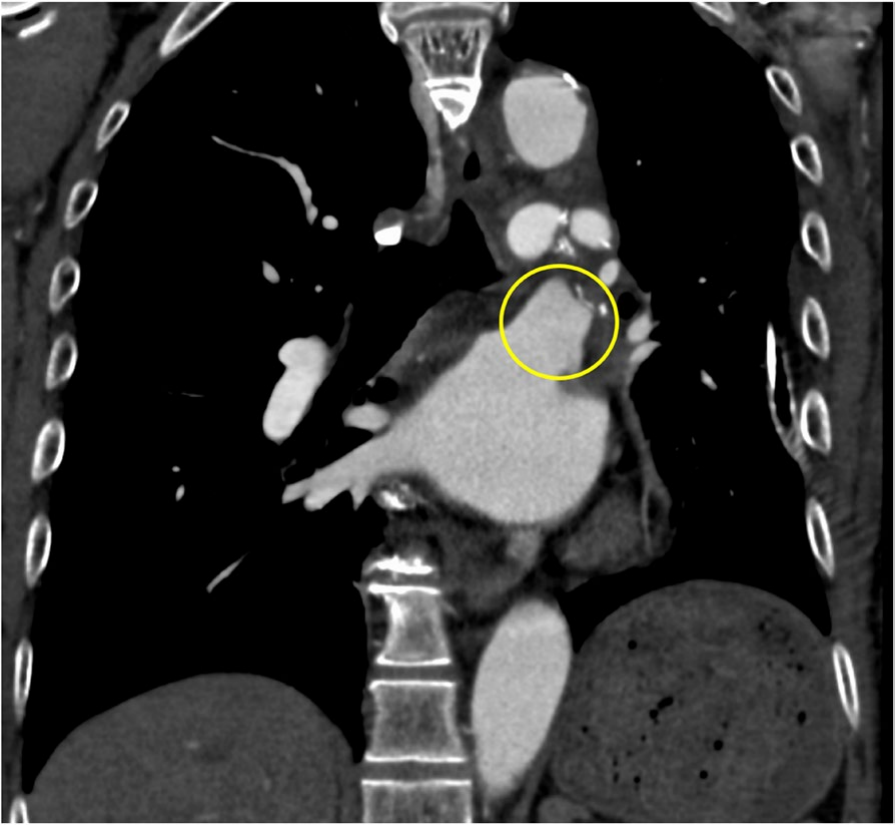
Contrast-enhanced chest computed tomography scan showing no thrombus in the stump of the left superior pulmonary vein (yellow circle)

**Fig. 4:**
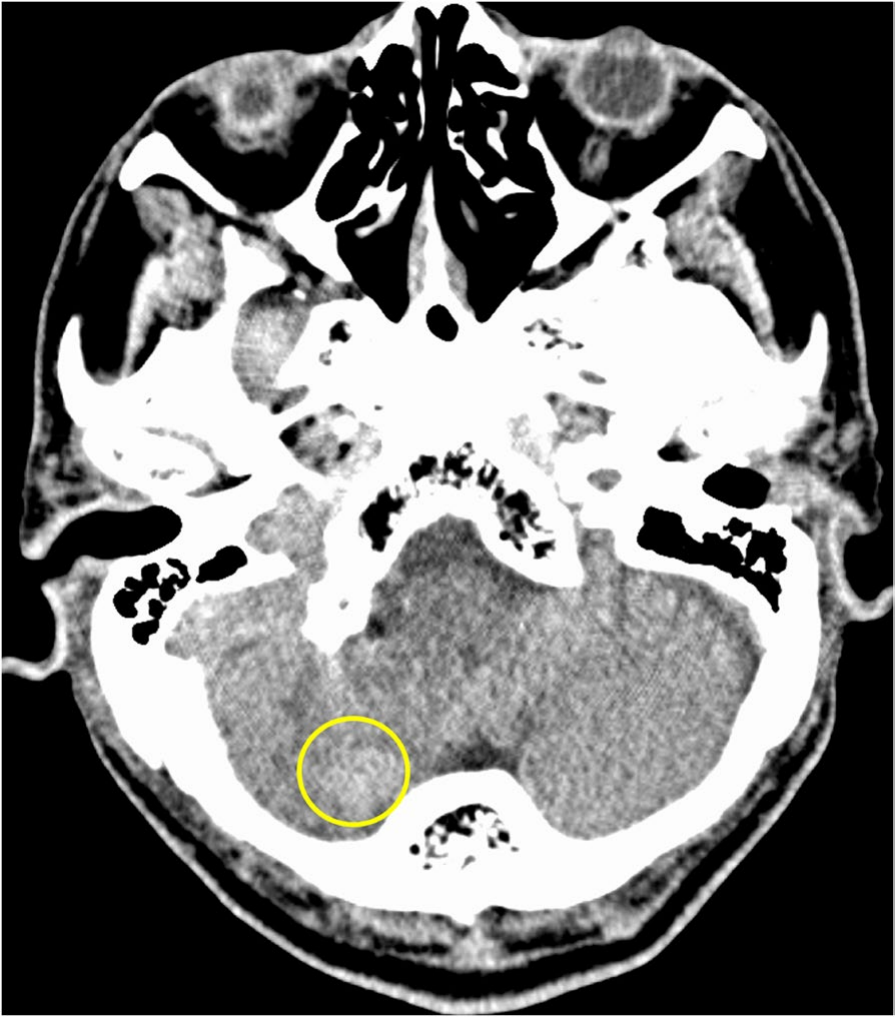
Brain computed tomography scan showing hemorrhagic infarction in the cerebellum on postoperative day 9 (yellow circle)

The patient underwent video-assisted thoracoscopic left upper lobectomy and upper mediastinal lymph node dissection (ND2a-1). The left superior pulmonary vein was divided by using a linear stapler. The patient developed atrial fibrillation on the day after surgery and was treated with landiolol hydrochloride and continuous intravenous heparin infusion. At noon on postoperative day 2, the patient complained of dizziness and dysphagia. While his consciousness level was Japan Coma Scale 0, speech disturbances, ptosis of the left labial angle, hoarseness, and ataxia of the right upper and lower limbs were noted. He had a National Institutes of Health Stroke Scale score of 3. The patient underwent contrast-enhanced chest CT and emergency magnetic resonance imaging after the removal of the epidural catheter. Magnetic resonance imaging indicated an early ischemic area on the right lateral side of the medulla oblongata and cerebellum (Fig. 2a). Magnetic resonance angiography revealed right vertebral artery occlusion, which was not seen preoperatively (Fig. 2b–d). Contrast-enhanced chest CT revealed no evidence of thrombus in the stump of the left superior pulmonary vein (Fig. 3). The patient was diagnosed with Wallenberg syndrome, which occurred in the posterior inferior cerebral artery, and anticoagulation therapy with heparin and edoxaban was started. The repetitive saliva swallowing score test value was 3 times/30 s, and videoendoscopic evaluation of swallowing indicated right vocal cord paralysis. On postoperative day 9, a follow-up brain CT showed hemorrhagic infarction in the cerebellum (Fig. 4), but no new adverse events occurred. The patient was discharged and transferred to another hospital for rehabilitation on postoperative day 16.

## Discussion and conclusions

This is a rare case of Wallenberg’s syndrome after pulmonary resection. The frequency of Wallenberg syndrome is estimated to be approximately 2% in acute cerebral infarction [1], and Wallenberg syndrome presents a variety of neurological abnormalities that are difficult to diagnose [2]. The main complaints of Wallenberg syndrome are similar to the side effects of general anesthesia, such as dizziness, postoperative nausea, and vomiting. However, ataxia and dysarthria do not usually appear as side effects of anesthesia, while dizziness and nausea suddenly appear in patients after general anesthesia, especially after the left upper lobectomy. Hence, special attention and detailed physical examination are required in cases of Wallenberg Syndrome.

A literature search was performed in January 2022 using PubMed, with the following terms: (“lung cancer” OR “NSCLC”) AND (“surgery” OR “lobectomy”) AND “Wallenberg.” We found 31 articles on the criteria worldwide; however, no case of Wallenberg syndrome after pulmonary resection was discovered. By searching with the terms: (“lung cancer” OR “NSCLC”) AND (“surgery” OR “lobectomy” OR “pulmonary resection”) AND “cerebral infarction,” we performed a literature review of cases of cerebral infarction after pulmonary resection, in which the site of cerebral infarction could be identified (Table 1) [3–13]. Most of them had a cerebral infarction in the region of the anterior circulation, such as the internal carotid artery and the middle cerebral artery, and only two cases were in the region of the posterior circulation. To the best of our knowledge, this is the first reported case of Wallenberg syndrome following pulmonary resection.

**Table 1:** Literature review of the cases of cerebral infarction after pulmonary resection

Reference number	Age	Sex	Procedures	Postoperative period (day)	Symptoms suspected cause of embolism	Causing	Location artery of embolism
3	76	M	LUL	2	Hemiplegia, facial nerve palsy	Atrial fibrillation	Right middle cerebral artery
3	67	F	LLL	4	Hemiplegia, aphasia, and somnolence	Unknown	Left middle cerebral artery
3	71	M	LUL	9	Hemiplegia and somnolence	Unknown	Right middle cerebral artery
3	58	M	LUL	4	Dysarthria and aphasia	Unknown	Right middle cerebral artery
4	58	M	LUL	2	Consciousness disorder, hemiplegia, and aphasia	PV stump	Left internal carotid artery
5	70	M	RUL	12	Consciousness disorder and hemiplegia	Unknown	Left middle cerebral artery
6	70	M	LUL	1	Unknown	PV stump	Right internal carotid artery
6	68	M	LUL	9	Unknown	PV stump	Right middle cerebral artery
6	55	M	LUL	3	Unknown	PV stump	Right vertebral artery
7	71	F	LUL	2	Left hemiparesis	Unknown	Right internal carotid artery
8	72	M	LUL	4	A comatose state	PV stump	Right vertebral artery
8	55	M	Rt. S7–10 seg	2	Left hemiparesis	PV stump	Right middle cerebral artery
8	73	M	LUL	2	Left hemiparesis	PV stump	Rt. middle cerebral artery
9	77	F	LUL	9	Left hemiplegia	Unknown	Right middle cerebral artery
9	71	F	Lt. S1–3 seg	4	Left hemiplegia	Unknown	Right internal carotid artery
10	66	M	LUL	2	Left limb paralysis	PV stump	Right internal carotid artery
11	77	F	LUL	8	Right upper limb paralysis and dysarthria	PV stump	Left middle cerebral artery
12	73	F	LUL	19	Left complete hemiplegia	PV stump	Right middle cerebral artery
13	76	M	LUL	3	Consciousness disorder and left hemiplegia	PV stump	Right internal carotid artery

*RUL* Right upper lobectomy, *LUL* Left upper lobectomy, *LLL* Left lower lobectomy, *seg* Segmentectomy, *PV* Pulmonary vein

The incidence of cerebral infarction after lung surgery is reported to be 0.3–0.6% [14, 15]. However, patients undergoing left upper lobectomy have a higher frequency of cerebral infarction than those undergoing resection of other lobes. Ohtaka et al. reported that thrombosis in the last superior pulmonary vein stump after left upper lobectomy was a common complication, and it was one of the causes of cerebral infarction [16]. Kimura et al. reported that patients with cerebral infarction after pulmonary resection experienced atrial fibrillation postoperatively at a high frequency [3]. In our case, no pulmonary vein stump thrombus was found on CT, but new atrial fibrillation occurred and returned to sinus rhythm before the onset of cerebral infarction. Although ultrasound assessment of carotid arteries and echocardiography was not performed in this case, a preoperative CT scan showed mild calcification in the right vertebral artery, suggesting stenosis at the same site compared with other arteries leading to the brain. We speculated that an embolus caused by atrial fibrillation lodged in the constricted right vertebral artery.

Generally, the administration of recombinant tissue plasminogen activator (rt-PA) must be considered a new acute cerebral infarction in hospitals [17]. However, in most patients after surgery, intravenous administration of rt-PA is contraindicated, particularly after pulmonary resection, because serious hemorrhagic complications may occur. Second, it was necessary to assess the possibility of endovascular treatment. One of the indications for endovascular treatment is acute occlusion of the internal carotid or middle cerebral artery; however, there have been no randomized controlled trials for vertebral artery occlusion. A meta-analysis of 17 patients who underwent endovascular treatment for vertebral artery occlusion showed a high resumption rate of 80% [18]. We hope safe endovascular treatment of the posterior circulatory system can be established.

In conclusion, we presented a rare case of Wallenberg syndrome after pulmonary resection in the posterior inferior cerebral artery area due to vertebral artery occlusion after lobectomy. Because cerebral infarction of the posterior circulation shows many similar symptoms due to the side effects of anesthetic drugs, careful physical examination is required to determine Wallenberg syndrome.

## Data Availability

Data sharing is not applicable to this article, as no datasets were generated or analyzed during the current study.

## Abbreviations

CT: Computed tomography
rt-PA: Recombinant tissue plasminogen activator
